# Therapeutic Efficacy of ^177^Lu-Labeled A20FMDV2 Peptides Targeting α_ν_β_6_

**DOI:** 10.3390/ph15020229

**Published:** 2022-02-15

**Authors:** Truc Thao Huynh, Sreeja Sreekumar, Cedric Mpoy, Buck Edward Rogers

**Affiliations:** 1 Department of Radiation Oncology, Washington University School of Medicine, St. Louis, MO 63108, USA; t.huynh@wustl.edu (T.T.H.); s.sreekumar@wustl.edu (S.S.); cmpoy@wustl.edu (C.M.); 2 Department of Chemistry, Washington University, St. Louis, MO 63130, USA

**Keywords:** integrin α_ν_β_6_, theranostics, albumin binder

## Abstract

Integrin α_ν_β_6_ promotes migration and invasion of cancer cells, and its overexpression often correlates with poor survival. Therefore, targeting α_ν_β_6_ with radioactive peptides would be beneficial for cancer imaging and therapy. Previous studies have successfully developed radiotracers based on the peptide A20FMDV2 that showed good binding specificity for α_ν_β_6_. However, one concern of these α_ν_β_6_ integrin-targeting probes is that their rapid blood clearance and low tumor uptake would preclude them from being used for therapeutic purposes. In this study, albumin binders were used to increase tumor uptake for therapeutic applications while the non-albumin peptide was evaluated as a potential positron emission tomography (PET) imaging agent. All peptides used the DOTA chelator for radiolabeling with either ^68^Ga for imaging or ^177^Lu for therapy. PET imaging with [^68^Ga]Ga-DOTA-(PEG28)_2_-A20FMDV2 revealed specific tumor uptake in α_ν_β_6_-positive tumors. Albumin-binding peptides EB-DOTA-(PEG28)_2_-A20FMDV2 and IBA-DOTA-(PEG28)_2_-A20FMDV2 were radiolabeled with ^177^Lu. Biodistribution studies in normal mice showed longer blood circulation times for the albumin binding peptides compared to the non-albumin peptide. Therapy studies in mice demonstrated that both ^177^Lu-labeled albumin binding peptides resulted in significant tumor growth inhibition. We believe these are the first studies to demonstrate the therapeutic efficacy of a radiolabeled peptide targeting an α_ν_β_6_-positive tumor.

## 1. Introduction

Integrins are an important class of cell surface receptors that are responsible for cell-matrix adhesion and signaling across the membrane, therefore controlling a variety of vital cell functions such as cellular growth, proliferation, migration, signaling, and cytokine activation that are critical to infection, inflammation, and cancer [1,2]. Their diverse functions make them attractive therapeutic targets and certain integrin-targeted drugs have been effectively utilized in the clinic or in clinical trials for cancer therapy [3,4,5,6]. In recent years, integrin α_ν_β_6_ has gained much attention due to its overexpression in various kinds of aggressive cancers and its correlation with worse prognosis and survival outcomes [7,8,9,10,11].

Several radiolabeled α_ν_β_6_-targeting ligands have been identified and used in preclinical imaging studies [12,13,14,15,16,17]. Quigley et al. introduced a cyclic nonapeptide c[YRGDLAYp(NMe)K] radiolabeled with ^68^Ga that showed a high affinity and target-specific uptake in integrin α_ν_β_6_-positive tumors [16]. Kimura et al. presented a series of highly stable cystine knot peptides radiolabeled with ^64^Cu that showed potent and specific integrin α_v_β_6_ binding in vitro and in vivo studies [17]. In this study, we focus on a 20-amino-acid peptide sequence of NAVPNLRGDLQVLAQKVART (A20FMDV2) reported by Hausner et al. that showed a high target affinity and selectivity for the integrin receptor [12]. Recently, this group translated a similar peptide in which the lysine at position 16 was replaced with an arginine (A20FMDV2-K16R) for clinical imaging when radiolabeled with ^18^F [18].

Similar to other peptides, A20FMDV2 cleared quickly from the blood circulation, resulting in poor tumor uptake and retention. The peptide was initially radiolabeled with ^18^F and in vivo studies in DX3puroβ6 tumor-bearing mice revealed a low tumor uptake of 0.66 ± 0.09% ID/g [12,15]. Hausner et al. then proposed the bi-terminal PEGylation of A20FMDV2 peptide that successfully resulted in a more favorable in vivo tumor uptake of 2.3 ± 0.2% ID/g in DX3puroβ6 tumor mouse models [15]. This led to a human imaging study of [^18^F]FBA-(PEG28)_2_-A20FMDV2-K16R, which proved the favorable performance of the peptide for the identification of small lesions in primary and metastatic sites [18]. In addition to PEGylation, efforts have been made to increase half-life through the attachment of peptides by non-covalent binding to blood components that have a long half-life such as albumin. This approach presents an advantage as it allows for the recycling of those proteins back into the blood and further extends the half-life of investigated peptides [19]. Hausner et al. recently reported A20FMDV2-K16R that incorporated a 4-(p-iodophenyl)butyric acid as an albumin binding moiety [14]. The peptide conjugated with NOTA and radiolabeled with aluminum [^18^F]fluoride demonstrated specificity in cell binding assays as well as increased blood circulation and tumor uptake in DX3puroβ6 tumor mouse models compared to the non-albumin binding peptide [14]. Ganguly et al. evaluated [^64^Cu]Cu-IP-DOTA-(PEG28)_2_-A20FMDV2-K16R (IP = albumin binding moiety) in mice bearing BxPC-3 tumors and showed 3-5 fold higher tumor uptake when compared to the non-albumin binding peptide [20].

**Figure 1:**
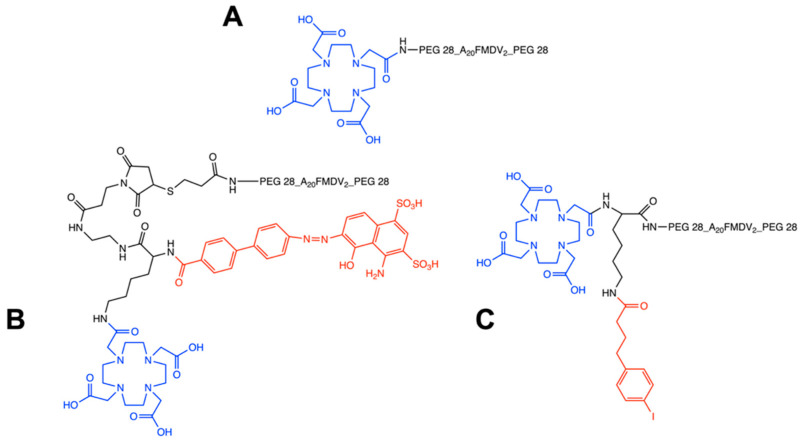
Chemical structures of non-albumin DOTA-(PEG28)_2_-A20FMDV2 (**A**), EB-DOTA-(PEG28)_2_-A20FMDV2 (**B**) and IBA-DOTA-(PEG28)_2_-A20FMDV2 (**C**). The DOTA chelator is indicated in blue, and albumin binders are indicated in red.

In the present studies, we focus on the bi-terminally PEGylated A20FMDV2 peptide conjugated with DOTA for the ^68^Ga and ^177^Lu radiolabeling (Figure 1). The non-albumin binding peptide was radiolabeled with ^68^Ga (T_1/2_ 68 min, β^+^ = 89%) and evaluated as a potential PET imaging agent. Two albumin binding peptides were developed that incorporated albumin binding moieties based on either 4-(p-iodophenyl)butyric acid (IBA) or Evans Blue azo dye. 4-(p-iodophenyl)butyric acid was reported by Dumelin et al. as one of the promising structures that formed stable non-covalent binding with serum albumin in the micromolar range [21]. On the other hand, Evans Blue (EB) is an albumin dye that can bind reversibly to serum albumin with IC_50_ in the micromolar range with each albumin binding to 14 molecules of EB [22]. Both albumin binders and their derivatives have been utilized for lymph node, tumor, and blood pool imaging due to their improved pharmacokinetics [20,23,24,25]. Our studies focused on the evaluation of two albumin-binding peptides EB-DOTA-(PEG28)_2_-A20FMDV2 and IBA-DOTA-(PEG28)_2_-A20FMDV2 radiolabeled with ^177^Lu (T_1/2_ = 6.7 d, Eβ^-^_avg_ = 134 keV) for their therapeutic potential with the main goal of using [^68^Ga]Ga-DOTA-(PEG28)_2_-A20FMDV2 and [^177^Lu]Lu-EB/IBA-DOTA-(PEG28)_2_-A20FMDV2 as a novel theranostic pair for the imaging and therapy of α_v_β_6_-positive tumors.

## 2. Results

### 2.1. Radiochemistry and Serum Stability

Non-albumin DOTA-(PEG28)_2_-A20FMDV2 was successfully radiolabeled with ^68^Ga with a radiochemical yield of >95% at the molar activity of 18.5 MBq/nmol (Figure S1). The two albumin peptides EB-DOTA-(PEG28)_2_-A20FMDV2 and IBA-DOTA-(PEG28)_2_-A20FMDV2 were readily labeled with ^177^Lu at the molar activity of 10 MBq/nmol. The radioligands showed high radiochemical purity of >98% as evaluated by radioTLCs (Figure S1). Both albumin-binding radioligands were >90% intact for up to 7 days in human serum (Figure S2).

### 2.2. Cellular Uptake and Internalization

**Figure 2:**
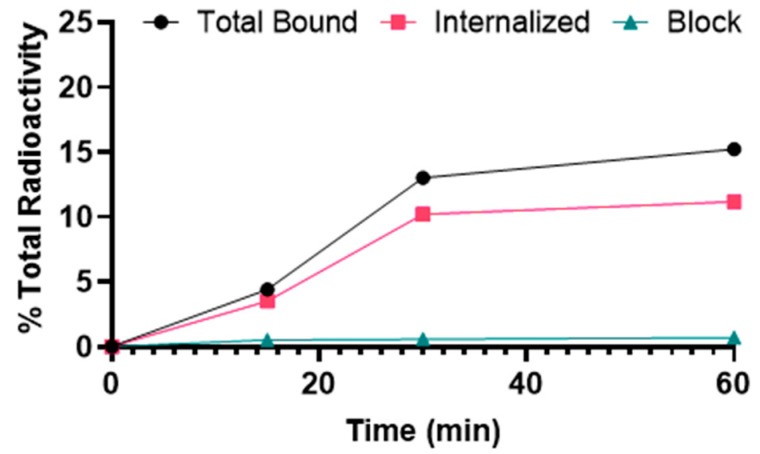
Binding and internalization curves of [^68^Ga]Ga-DOTA-(PEG28)_2_-A20FMDV2 in the BxPC-3 cell line. Total bound and internalization levels were shown as a percentage relative to the total radioactivity added. Data were presented as triplicates of mean ± SD.

[^68^Ga]Ga-DOTA-(PEG28)_2_-A20FMDV2 showed binding to α_v_β_6_–expressing BxPC-3 cells that was significantly inhibited by blocking with A20FMDV2, demonstrating α_v_β_6_-specific binding (Figure 2). The uptake of [^68^Ga]Ga-DOTA-(PEG28)_2_-A20FMDV2 was 4.4 ± 0.2% at 15 min after addition of the radiotracer and increased gradually over time to 15.2 ± 0.2% at 1 h. The internalized fraction was 3.5 ± 0.4% after 15 min of incubation, increased to 11.2 ± 0.4% after 1 h.

**Figure 3:**
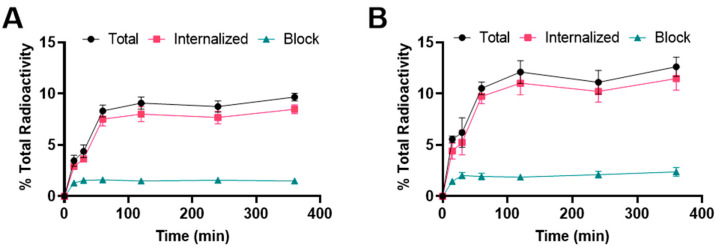
Binding and internalization curves of (**A**) [^177^Lu]Lu-EB-DOTA-(PEG28)_2_-A20FMDV2 or (**B**) [^177^Lu]Lu-IBA-DOTA-(PEG28)_2_-A20FMDV2 in BxPC-3 cell line. Total bound and internalization levels were shown as a percentage relative to the total radioactivity. Data were presented as triplicates of mean ± SD.

Binding and internalization curves indicated the specific binding of ^177^Lu radiolabeled albumin peptides towards integrin α_v_β_6_ in BxPC-3 cells (Figure 3). Binding was specific as the addition of the blocking agent reduced binding to <3%. For [^177^Lu]Lu-EB-DOTA-(PEG28)_2_-A20FMDV2, the uptake was 3.5 ± 0.5% at 15 min and increased to 8.3 ± 0.6% at 1 h where it remained about the same for subsequent time points. The internalized fraction accounted for more than 80% of the total bound radioactivity. For [^177^Lu]Lu-IBA-DOTA-(PEG28)_2_-A20FMDV2, the uptake was 5.6 ± 0.3% at 15 min and increased to 10.5 ± 0.6% at 1 h where it remained about the same for subsequent time points. More than 80% of the total bound radioactivity was internalized into the cells. The [^177^Lu]Lu-IBA-DOTA-(PEG28)_2_-A20FMDV2 also showed a slightly higher binding and internalization than [^177^Lu]Lu-EB-DOTA-(PEG28)_2_-A20FMDV2 in the BxPC-3 cell line, but both constructs were slightly lower than [^68^Ga]Ga-DOTA-(PEG28)_2_-A20FMDV2 at 1 h.

### 2.3. ^68^Ga Imaging Studies

**Figure 4:**
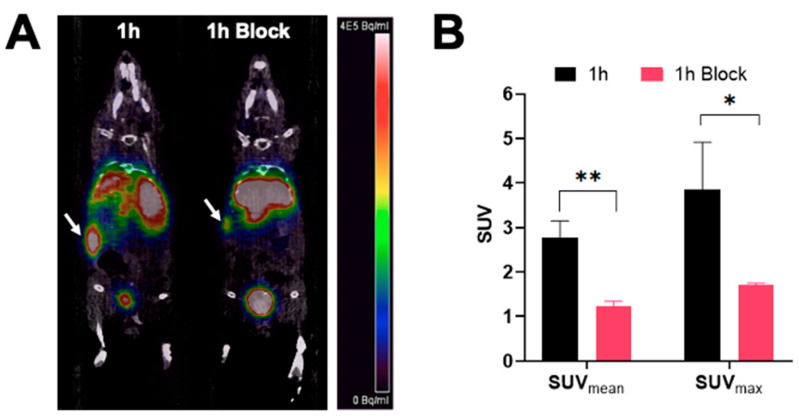
PET imaging of [^68^Ga]Ga-DOTA-(PEG28)_2_-A20FMDV2 in athymic nude mice bearing BxPC-3 tumors (**A**) Representative micro-PET/CT co-registration images of a coronal section at 1 h after intravenous injection of 3.7 MBq of [^68^Ga]Ga-DOTA-(PEG28)2-A20FMDV2 with or without blocking (*n* = 3). White arrows indicate the positions of the tumor xenografts. The scale bar unit is Bq/mL (**B**) Mean and maximum standard uptake values of imaged tumors (* *p* < 0.05, ** *p* < 0.01).

PET/CT images showing a coronal section of mice after injection with [^68^Ga]Ga-DOTA-(PEG28)_2_-A20FMDV2 at 1 h are shown in Figure 4. The image clearly shows tumor accumulation of [^68^Ga]Ga-DOTA-(PEG28)_2_-A20FMDV2 at 1 h post-injection that was inhibited by an excess of blocking agent. Image analysis of the tumors shows that mice receiving blocking agent had significantly smaller standard uptake values, SUVs (SUV_mean_: 1.22 ± 0.12, SUV_max_: 1.71 ± 0.04) compared to the non-block mice (SUV_mean_: 2.77 ± 0.38, SUV_max_: 3.85 ± 1.06).

### 2.4. ^177^Lu Biodistribution Studies

To assess the effect of albumin binding motifs in improving blood circulation half-life, CD-1 mice were injected with either 0.37 MBq of [^177^Lu]Lu-DOTA-(PEG28)_2_-A20FMDV2, [^177^Lu]Lu-EB-DOTA-(PEG28)_2_-A20FMDV2, or [^177^Lu]Lu-IBA-DOTA-(PEG28)_2_-A20FMDV2 (Figure S3). Significantly more blood uptake was observed for the albumin binders at 1 h with 5.36 ± 1.06% ID/g for [^177^Lu]Lu-EB-DOTA-(PEG28)_2_-A20FMDV2 and 4.70 ± 0.68% ID/g for [^177^Lu]Lu-IBA-DOTA-(PEG28)_2_-A20FMDV2 compared to 0.11 ± 0.04% ID/g for [^177^Lu]Lu-DOTA-(PEG28)_2_-A20FMDV2 (*p* < 0.00001) (Figure S3E). The albumin binding peptides cleared from the blood over time, as evidenced in 0.41 ± 0.03% ID/g for [^177^Lu]Lu-EB-DOTA-(PEG28)_2_-A20FMDV2 and 0.09 ± 0.02% ID/g for [^177^Lu]Lu-IBA-DOTA-(PEG28)_2_-A20FMDV2 at 48 h p.i. Increased radioactivity accumulation in off-target organs was observed in the albumin-binding peptides when compared to the non-albumin peptide in the lung, liver, spleen, heart, and muscle (Figure S3A–C). In the case of the kidney, although [^177^Lu]Lu-IBA-DOTA-(PEG28)_2_-A20FMDV2 showed uptake of 93.40 ± 13.46% ID/g at 1 h, it cleared rapidly resulting in 24.97 ± 1.92% ID/g at 48 h, which was lower than the kidney uptake observed by non-albumin DOTA-(PEG28)_2_-A20FMDV2 (34.72 ± 13.20% ID/g at 48 h) (Figure S3D). On the other hand, [^177^Lu]Lu-EB-DOTA-(PEG28)_2_-A20FMDV2 showed higher and more persistent levels of radioactivity in the kidney than non-albumin counterpart at all time points (44.26 ± 5.13% ID/g at 48 h).

**Figure 5:**
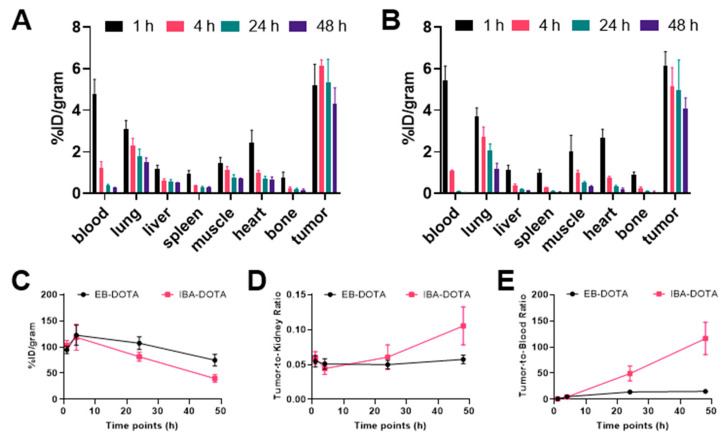
Biodistribution of ^177^Lu albumin binding radiotracers in mice bearing α_v_β_6_-expressing BxPC-3 xenograft tumors (**A**) Radiotracer uptake of [^177^Lu]Lu-EB-DOTA-(PEG28)_2_-A20FMDV2 (**B**) Radiotracer uptake of [^177^Lu]Lu-IBA-DOTA-(PEG28)_2_-A20FMDV2 in tumors and selected organs (%ID/g; bars = SD; tumors: *n* = 4–5 per time point) (**C**) Kidney uptake of ^177^Lu albumin binding radiotracers (**D**) Tumor to kidney ratios (**E**) Tumor to blood ratios.

Figure 5 shows the biodistribution of [^177^Lu]Lu-EB-DOTA-(PEG28)_2_-A20FMDV2 and [^177^Lu]Lu-IBA-DOTA-(PEG28)_2_-A20FMDV2 in mice bearing BxPC-3 tumor xenografts. Tumor uptake of [^177^Lu]Lu-EB-DOTA-(PEG28)_2_-A20FMDV2 was 5.20 ± 1.02% ID/g at 1 h p.i, and remained relatively constant at subsequent time points. Uptake in the blood was 4.80 ± 0.69% ID/g at 1 h that decreased to 0.28 ± 0.01% ID/g at 48 h p.i. For [^177^Lu]Lu-IBA-DOTA-(PEG28)_2_-A20FMDV2, tumor uptake was 6.12 ± 0.70% ID/g, and dropped slightly to 4.06 ± 0.54% ID/g at 48 h p.i. Blood uptake was 5.43 ± 0.71% ID/g at 1 h p.i, followed by rapid clearance to 0.04 ± 0.01% ID/g at 48 h p.i. Similar to normal mice, kidney uptake was high with the IBA construct clearing more rapidly than the EB construct. Uptake in normal organs such as lung, liver, spleen, muscle, heart, and bone was low with less than 4% ID/g at all time points.

When comparing the two albumin binding peptides, uptake in tumor and normal tissues was similar at early time points (Tables S1 and S2). However, at 24 h and 48 h, [^177^Lu]Lu-IBA-DOTA-(PEG28)_2_-A20FMDV2 also cleared more rapidly from the blood, as evidenced by more than a 7-fold higher tumor-to-blood ratio at 48 h p.i when tumor uptake remained relatively similar (4.29 ± 0.78% ID/g for [^177^Lu]Lu-EB-DOTA-(PEG28)_2_-A20FMDV2 and 4.06 ± 0.54% ID/g for [^177^Lu]Lu-IBA-DOTA-(PEG28)_2_-A20FMDV2, respectively) (Figure 5E).

### 2.5. Therapy Studies

The anti-tumor efficacy was investigated in BxPC-3 tumor-bearing mice with a single dose of 37 MBq [^177^Lu]Lu-EB-DOTA-(PEG28)_2_-A20FMDV2 or [^177^Lu]Lu-IBA-DOTA-(PEG28)_2_-A20FMDV2 (Figure S4). Tumor volumes were found to be significantly reduced in comparison to the control group (*p* < 0.001) for both treated groups (Figure S4). However, treated mice experienced significant weight loss of more than 20%, resulting in the death of all mice from [^177^Lu]Lu-EB-DOTA-(PEG28)_2_-A20FMDV2 two weeks after radiotracer injection. Mice treated with [^177^Lu]Lu-IBA-DOTA-(PEG28)_2_-A20FMDV2 also showed significant weight loss, but not as severe as the [^177^Lu]Lu-EB-DOTA-(PEG28)_2_-A20FMDV2 counterpart.

**Figure 6:**
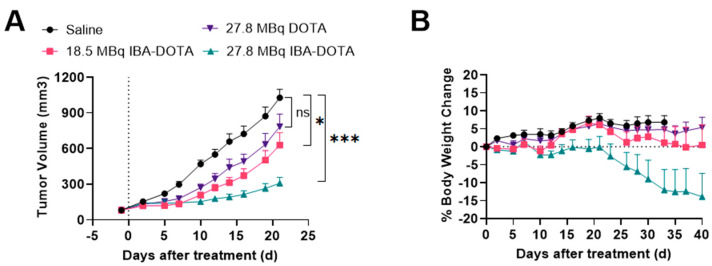
Inhibition of BxPC-3 tumor growth (*n* = 8) and body weight changes following treatment with saline, 18.5 MBq or 27.8 MBq [^177^Lu]Lu-IBA-DOTA-(PEG28)_2_-A20FMDV2 or 27.8 MBq [^177^Lu]Lu-DOTA-(PEG28)_2_-A20FMDV2 (**A**) Tumor curves until the last day all mice remained in the study (day 23) (* *p* < 0.05, *** *p* < 0.001) (**B**) Body weight changes (±SEM) with the dotted line indicating baseline. The data represents the mean percent weight change from baseline (day 1) for each group.

Based on these results, we performed a subsequent therapy study which focused on [^177^Lu]-IBA-DOTA-(PEG28)_2_-A20FMDV2 since it demonstrated less toxicity compared to [^177^Lu]-EB-DOTA-(PEG28)_2_-A20FMDV2. A therapy study with reduced doses of [^177^Lu]Lu-IBA-DOTA-(PEG28)_2_-A20FMDV2 was performed in comparison with non-albumin [^177^Lu]Lu-DOTA-(PEG28)_2_-A20FMDV2 and saline as a control (Figure 6). There was no significant weight loss in mice from the control and [^177^Lu]Lu-DOTA-(PEG28)_2_-A20FMDV2 treated groups during the course of the study. One mouse treated with 18.5 MBq of [^177^Lu]Lu-IBA-DOTA-(PEG28)_2_-A20FMDV2 and 4 out of 8 mice for 27.8 MBq of [^177^Lu]Lu-IBA-DOTA-(PEG28)_2_-A20FMDV2 experienced significant weight loss of greater than 20% of body weight. Mice experienced weight loss at delayed time points when injected with reduced doses compared to the first therapy study. Tumor curves were compared from the day of injection until the last day when all mice were alive (day 23) (Figure 6A). Therapy with non-albumin [^177^Lu]Lu-DOTA-(PEG28)_2_-A20FMDV2 did not show significant tumor growth inhibition compared to the control mice. Mice treated with [^177^Lu]Lu-IBA-DOTA-(PEG28)_2_-A20FMDV2 showed tumor inhibition, and the relative tumor volume of the 27.8 MBq group was found to be lower than that observed in the 18.5 MBq group (*p* = 0.04). Both [^177^Lu]Lu-IBA-DOTA-(PEG28)_2_-A20FMDV2 treated groups showed effective tumor inhibition in comparison to control group (*p* < 0.01 and *p* < 0.001 for 18.5 Mbq and 27.8 MBq dose, respectively).

### 2.6. Immunohistochemical Staining

A review of hematoxylin-eosin (H&E) stained tissue samples revealed that kidney injury was associated with the administration of high-dose [^177^Lu]Lu-IBA-DOTA-(PEG28)_2_-A20FMDV2 (37 MBq per mouse) (Figure S5). The morphological changes suggested tubular epithelial degeneration and necrosis as presented in the increased amount of casts and dilated tubules lacking brush borders. Initial examination of the Ki-67-stained tumor tissues showed a significant decrease in Ki-67 expression for the 37 MBq [^177^Lu]Lu-IBA-DOTA-(PEG28)_2_-A20FMDV2 treated group (34.51 ± 5.24%) when compared to the control group (51.39 ± 5.56%) (*p* = 0.0044). Injury was not observed in the heart, lung, liver and spleen sections (data not shown).

## 3. Discussion

There is a wide prevalence of α_v_β_6_-integrin expression in different kinds of cancer. It is known that an elevated level of integrin α_v_β_6_ is associated with poor prognosis as it promotes cell invasion and migration—the two crucial processes responsible for metastasis. In recent years, a number of radiolabeled α_v_β_6_ integrin ligands for in vivo imaging and therapy of the integrin have been developed. Among these, the 20-mer peptide, A20FMDV2, has been extensively studied and radiolabeled with a variety of radionuclides (^18^F, ^68^Ga, ^64^Cu, and ^111^In) [26,27,28,29]. However, it has not been evaluated as of yet with therapeutic radionuclides, likely because of its short blood half-life that leads to low tumor uptake.

One of the most promising approaches to increase blood half-life is the incorporation of albumin-binding molecules that can form noncovalent, reversible interactions with serum albumin and prolong the in vivo blood circulatory half-life of conjugated peptides. The combination of albumin-binding moieties derived from Evans Blue dye and 4-(p-iodophenyl)butyric acid with targeted radiopharmaceuticals demonstrates prolonged blood circulation and increased tumor uptake. The conjugation with truncated EB resulted in improved tumor uptake and pharmacokinetics using ^177^Lu-labeled tumor targeting vectors specific for somatostatin receptors (EB-TATE), integrin α_v_β_3_ (EB-cRGD), prostate-specific membrane antigen (EB-PSMA-617), and glucagon-like peptide-1 receptor (EB-exendin-4) [24,30,31,32]. On the other hand, the introduction of a 4-(p-iodophenyl)butyric acid derivatives showed promising in vivo results using [^177^Lu]Lu-folates-cm10 for radionuclide therapy of folate receptor α (FR)-positive cancer and [^177^Lu]-PSMA-ALB-2 for prostate cancer therapy [33,34]. The present study compares the two albumin-binding peptides—EB-DOTA-(PEG28)_2_-A20FMDV2 and IBA-DOTA-(PEG28)_2_-A20FMDV2 for in vivo therapy of α_v_β_6_-positive BxPC-3 tumors when radiolabeled with ^177^Lu.

All radiotracers were radiolabeled in good radiochemical purity at a molar activity of 10–18.5 MBq/nmol. A high temperature of 90 °C was required for the labeling, which is consistent with previous studies, which used up to 99 °C for ^68^Ga and 80–95 °C for ^177^Lu labelings [35,36,37]. In vitro cell-based studies in BxPC-3 cell lines indicated that about 11% internalized for the [^68^Ga]Ga-DOTA-(PEG28)_2_-A20FMDV2 at 1 h, while the albumin binding constructs had about 8-10%. These results are similar to what was observed for the ^64^Cu-labeled constructs evaluated by Ganguly et al., in which 14.5% and 11.9% internalization was observed for their non-albumin and albumin binding peptides, respectively, in BXPC-3 cells [20]. Of course, a direct comparison is difficult due to different radionuclides, linkers, and the K16R substitution. We demonstrated good imaging of BXPC-3 tumors with [^68^Ga]Ga-DOTA-(PEG28)_2_-A20FMDV2 that was specific as evidenced by the reduction in SUV upon administration of the blocking agent. Other ^68^Ga studies targeting integrin α_v_β_6_ have shown good tumor uptake compared to blocking and clearance through the kidney, which is consistent with our results [16,35,36,38].

In vivo studies in CD-1 mice revealed prolonged blood circulation of the albumin binding peptides, but also higher accumulation in non-target organs. This phenomenon is due to the longer retention of radiolabeled compounds in the bloodstream. A previous study that examined the effect of albumin binder on somatostatin peptide analogs also revealed significantly greater uptake of the modified Evans Blue compound [^177^Lu]Lu-DMEB-TATE in the normal tissues at all time points compared to the uptake of the non-albumin [^177^Lu]Lu-DOTA-TATE counterpart [24]. Biodistribution studies in BxPC-3 tumor-bearing mice confirmed the prolonged half-life in blood as a result of low micromolar affinity to albumin, in which the blood uptake at 4 h was 1.23 ± 0.30% ID/g for [^177^Lu]Lu-EB-DOTA-(PEG28)_2_-A20FMDV2 and 1.06 ± 0.07% ID/g for [^177^Lu]Lu-IBA-DOTA-(PEG28)_2_-A20FMDV2. The blood uptake for [^177^Lu]Lu-IBA-DOTA-(PEG28)_2_-A20FMDV2 was less than [^64^Cu]Cu-IP-DOTA-(PEG28)_2_-A20FMDV2-K16R (2.42 ± 0.15% ID/g at 4 h), and both showed rapid blood clearance after the initial time point [20]. BxPC-3 tumor uptake of [^177^Lu]Lu-IBA-DOTA-(PEG28)_2_-A20FMDV2 reduced slightly over time (5.14 ± 0.92% ID/g at 4 h to 4.06 ± 0.54% ID/g at 48 h), which is consistent with [^64^Cu]Cu-IP-DOTA-(PEG28)_2_-A20FMDV2-K16R (5.93 ± 0.60% ID/g at 4 h to 4.90 ± 0.57% ID/g at 48 h). Kidney uptake of [^177^Lu]Lu-IBA-DOTA-(PEG28)_2_-A20FMDV2 (118.64 ± 24.54% ID/g at 4 h) was significantly higher than that of [^64^Cu]Cu-IP-DOTA-(PEG28)_2_-A20FMDV2-K16R (23.06 ± 2.31% ID/g at 4 h) [20]. Again, similar to the in vitro studies, the difference between our data and the results observed by Ganguly et al. may be due to the radionuclide used, the linker, or the K16R mutation [20].

Therapy studies showed that both [^177^Lu]Lu-EB-DOTA-(PEG28)_2_-A20FMDV2 and [^177^Lu]Lu-IBA-DOTA-(PEG28)_2_-A20FMDV2 led to a significant tumor inhibition over the course of the study. However, increased toxicity was observed for [^177^Lu]Lu-EB-DOTA-(PEG28)_2_-A20FMDV2, which correlates to the increased kidney uptake at later time points observed with this construct compared to the [^177^Lu]Lu-IBA-DOTA-(PEG28)_2_-A20FMDV2 as discussed above. Histological analysis of the kidney demonstrated morphological changes indicating toxicity to this organ. The therapeutic effect was greatly enhanced upon the incorporation of the albumin binder when compared with that of the non-albumin [^177^Lu]Lu-DOTA-(PEG28)_2_-A20FMDV2. Reducing the amount of radioactivity administered alleviated some toxicity that was seen with the 37 MBq dose while still producing a therapeutic response; however, tumor to normal tissue ratios must be improved in order to increase the therapeutic index. Therefore, further modifications to the peptide must be made to reduce the normal tissue uptake, especially for the kidney.

## 4. Materials and Methods

### 4.1. General Methods

All solvents and reagents were purchased from Sigma-Aldrich (St. Louis, MO, USA) or Fisher Scientific (Pittsburgh, PA, USA) and used as received. All solutions and buffers were prepared using HPLC-grade water. Radio-TLCs employed Whatman 60 Å silica gel thin-layer chromatography (TLC) plates and were analyzed using a Bioscan 200 imaging scanner (Bioscan, Inc., Washington, DC, USA). Radioactivity was counted with a Beckman Gamma 8000 counter containing a NaI crystal (Beckman Instruments, Inc., Irvine, CA, USA). The peptides EB-DOTA-(PEG28)_2_-A20FMDV2, IBA-DOTA-(PEG28)_2_-A20FMDV2, and non-albumin peptide DOTA-(PEG28)_2_-A20FMDV2 were synthesized by AnaSpec company (Fremont, CA, USA) and characterized by HPLC and mass spectrometry. A stock solution of 1 nmol/μL was made with HPLC-grade water and stored at −20 °C before use. Non-PEGylated A20FMDV2 peptide served as a blocking agent. BxPC-3 cells were purchased from ATCC (Manassas, VA, USA) and grown in RPMI 1640, 10% FBS, and 10 mM HEPES. The cells were cultured in an incubator at 37 °C, 5% CO2, and harvested in PBS by trypsin-EDTA 0.25% before use.

### 4.2. ^68^Ga Radiochemistry

^68^Ga was obtained from ^68^Ge/^68^Ga generator (Eckert and Ziegler) at Mallinckrodt Institute of Radiology, Washington University School of Medicine [39]. Briefly, ^68^Ga was eluted from the generator in 5mL of 0.1M HCl and collected into a vial. The resulting solution was loaded onto a strong cation Strata XC column 30 mg/mL 33 μm (Phenomenex) and the activity was retained in the column. The column was eluted with 1 mL 98% acetone (0.02M HCl) and the resulting activity was collected in a 1.5 mL Eppendorf tube. The solution was heated to evaporation at 90 °C for 15 min until 10–20 µL ^68^Ga was achieved. Aqueous ammonium acetate (0.1 M, pH 5.5, 100 µL) and a solution of DOTA-(PEG28)_2_-A20FMDV2 (2 nmol) were added to a solution of 1 mCi ^68^Ga. The reaction mixture was incubated at 90 °C for 15 min and evaluated for radiochemical purity by thin-layer chromatography with the mobile phase of 50 mM DTPA. The radiolabeled complex remained at the origin while the free ^68^Ga moved with the solvent front.

### 4.3. ^177^Lu Radiochemistry

^177^Lu (no-carrier added) was obtained from the University of Missouri Research Reactor (MURR). For ^177^Lu labelings, ^177^LuCl_3_ in 0.05 M HCl was mixed with NH_4_OAc 0.1M pH 5.5 in a 10:1 ratio to obtain a solution of pH 5.0–5.5. After the addition of the albumin conjugates (4–5 μL stock solution) to 0.5 mCi of ^177^Lu, the reaction vial was incubated for 15 min at 90 °C. For therapy studies, 100 μL stock solution of albumin peptides were added to 10 mCi of ^177^Lu. Quality control was performed by TLC as described above. The radiolabeled complexes remain at the origin while free ^177^Lu moves with the solvent front. Radiolabeled products (>95% purity) were used directly without further purification.

### 4.4. Serum Stability

The stability of the radioligands was determined over time using radioTLCs with 50 mM DTPA as mobile phase. 10 μL of radiotracers was added to the Eppendorf tubes, each containing 100 μL of human serum or 1X PBS. The tubes were incubated at 37 °C with moderate agitation. The integrity of the compounds was investigated after incubation at 1, 3, 5, and 7 days. The experiment was conducted in triplicates and 0.5 μL aliquots of radiotracers were to be withdrawn to evaluate the amount of intact compound by TLC.

### 4.5. Cellular Uptake and Internalization

The total cell binding and internalized fractions were conducted using α_ν_β_6_-positive human pancreatic BxPC-3 cell line. The cells were harvested in PBS 1X containing 0.05% (*w/v*) bovine serum albumin at a density of 20 × 10^6^ cells/mL. For [^68^Ga]Ga-DOTA-(PEG28)_2_-A20FMDV2, 3.7 kBq aliquots diluted in 10 μL PBS 1X were added to each tube containing 1 million cells suspended in 50 μL PBS (0.05% BSA). The cells were incubated for 15, 30, and 60 min at 37 °C with moderate shaking to prevent the settling of cells. For [^177^Lu]Lu-EB-DOTA-(PEG28)_2_-A20FMDV2 or [^177^Lu]Lu-IBA-DOTA-(PEG28)_2_-A20FMDV2, 3.7 kBq aliquots were added and the cells were incubated for 15, 30, 60, 120, 240 min, and 360 min. At each time point, the cells were washed with ice-cold PBS twice and cell pellets were counted for total binding activity on a gamma counter. For internalization assays, after the removal of the supernatant at the indicated time points, 20 mM sodium acetate (pH 4.0) was added to the cells to remove surface-bound radioactivity. The resulting cells were incubated at room temperature for 5 min, and the acid buffers were removed. Cells were then rinsed twice with ice-cold PBS and cell pellets were collected and counted for the activity of internalized fraction. In parallel to each experiment, blocking studies with an excess of 20 μg non-PEGylated peptide was conducted. The experiments were performed in triplicates.

### 4.6. Biodistribution Studies

Animals were supplied from Charles River Laboratories (Wilmington, MA, USA), and were handled in compliance with the Guidelines for Care and Use of Research Animals established by the Division of Comparative Medicine and the Animal Studies Committee of Washington University School of Medicine. Biodistribution studies were conducted in athymic nude mice bearing subcutaneous BxPC-3 tumors. Tumors were implanted on the right flank with 5 × 10^6^ cells about 4 weeks before the performance of the experiments. Biodistribution studies were performed when the tumors reached approximately 5–7 mm in diameter. Mice (*n* = 4–5) were injected intravenously with 0.37 MBq (10 µCi) of [^177^Lu]Lu-EB-DOTA-(PEG28)_2_-A20FMDV2 or [^177^Lu]Lu-IBA-DOTA-(PEG28)_2_-A20FMDV2 diluted in 100 μL saline and sacrificed by cervical dislocation at 1, 4, 24 and 48 h. Tissues of interest (blood, lung, liver, spleen, kidney, muscle, heart, bone, and tumor) were collected and weighed, and the radioactivity was measured using a gamma counter. The results were expressed as the percentage of injected dose per gram of tissue (%ID/g).

The comparison of non-albumin binding and albumin binding peptides were evaluated in CD-1 mice at 1, 4, 24, and 48 h. ^177^Lu-labeled peptides were prepared at the specific activity of 10 MBq/nmol. Mice (*n* = 4–5) were intravenously injected with 0.37 MBq (10 µCi) of respective radioligands diluted in 100 μL saline and sacrificed at specified time points. Selected organs were collected, weighed, and counted for activity. The results were reported as the percentage of the injected radioactivity per gram of tissue mass (% ID/g) and the radioactivity was calibrated using a known standard.

### 4.7. PET Imaging Studies

Mice (*n* = 3) were injected with 3.7 MBq (100 µCi) of [^68^Ga]Ga-DOTA-(PEG28)_2_-A20FMDV2 and PET imaging was performed 1 h after radiotracer injection. For blocking study, excess of unlabeled peptides was administered 1–2 min before radiotracer injection. The PET scans were acquired for 20 min on an Inveon small animal PET/CT scanner (Siemens Medical Solutions, Malvern, PA, USA). Static images were reconstructed with the maximum a posteriori (MAP) reconstruction algorithm and corrected for decay. Image analysis was performed using the Inveon Research Workstation image display software (Siemens). Regions of interest (ROI) were selected based on co-registered anatomical CT images, and the average or maximum standard uptake value (SUV) was calculated as the mean or maximum regional radioactivity concentration (nCi/cc) x animal weight (g)/decay-corrected amount of injected dose (nCi).

### 4.8. Therapy Studies

Therapy studies were conducted in athymic nude mice bearing BxPC-3 tumors when the tumor volume reached about 100 mm^3^. An initial therapy study was conducted with control mice (*n* = 8) injected with saline and treated mice received either 37 MBq of [^177^Lu]Lu-EB-DOTA-(PEG28)_2_-A20FMDV2 or [^177^Lu]Lu-IBA-DOTA-(PEG28)_2_-A20FMDV2. Tumor volumes and body weights were monitored three times a week. Individual tumor size was calculated using the formula (length × width × width)/2. The mice were euthanized when the tumor reached 1500 mm^3^, ulceration of >4 mm was present, or significant stress due to weight loss. A second therapy study was conducted in BxPC-3 tumor-bearing mice in which mice (*n* = 8) were given either saline, 18.5 MBq [^177^Lu]Lu-IBA-DOTA-(PEG28)_2_-A20FMDV2, 27.8 MBq [^177^Lu]Lu-IBA-DOTA-(PEG28)_2_-A20FMDV2 or 27.8 [^177^Lu]Lu-DOTA-(PEG28)_2_-A20FMDV2. The doses were administered via tail vein and tumor size and mouse weights were recorded three times a week. Tumor volumes were plotted versus time to determine tumor growth inhibition.

### 4.9. Immunohistochemical Staining

Tumor, kidney, heart, lung, liver, and spleen of mice (*n* = 4) given either saline or 37 MBq dose of [^177^Lu]Lu-IBA-DOTA-(PEG28)_2_-A20FMDV2 were collected and fixed in neutral buffered formalin. After fixation and dehydration, tissue samples were embedded in paraffin, and 5 µm tissue sections were then stained with hematoxylin and eosin (H&E). H&E staining of tissue samples was prepared by the Anatomic and Molecular Pathology Core Lab, Washington University in St Louis. Tumors were also stained with Ki-67 for further microscopic examination. Before staining, slides were baked in 55 °C oven for 60 min to deparaffinize and heat-induced antigen retrieval was performed in citrate buffer 0.1 M pH 6.0 at 92 °C for 20 min to recover the antigens that may have been altered by fixation. Tissues were first blocked with Dako Endogenous Enzyme Block (Dako North America Inc., Carpinteria, CA, USA) for 10 min, followed by another blocking step with 10% goat serum in PBS for another 45 min. A primary antibody Ki-67 (9027, Cell Signaling, 1:100 dilution) was applied to the slides overnight at 4 °C. The secondary antibody ImmPRESS Goat Anti-rabbit (Vector Laboratories Inc., Mountain View, CA, USA) was then added for 45 min. The color was developed using DAB substrate Chromogen (Dako) and the sections were counterstained with Hematoxylin to visualize nuclei and overall tissue architecture. Sections were dehydrated, mounted, and cover-slipped. Staining results were assessed by Olympus microscope (BX51) and the cell Sense software.

### 4.10. Statistical Analysis

Quantitative data were processed by Prism 9 (GraphPad Software, La Jolla, CA, USA) and expressed as Mean ± SD. For therapy studies, tumor measurements and body weight changes were expressed as Mean ± SEM. Statistical analysis was performed using Student’s *t*-test. Differences at the 95% confidence level (*p* < 0.05) were considered statistically significant.

## 5. Conclusions

Here, we demonstrate the first targeted radiopharmaceutical therapy for tumors expressing α_v_β_6_ integrin. [^68^Ga]Ga-DOTA-(PEG28)_2_-A20FMDV2 and [^177^Lu]Lu-IBA-DOTA-(PEG28)_2_-A20FMDV2 could be used as a potential theranostic pair for imaging and therapy of α_v_β_6_-expressing tumors. [^68^Ga]Ga-DOTA-(PEG28)_2_-A20FMDV2 showed good tumor uptake and imaging of BxPC-3 tumors at 1 h. [^177^Lu]Lu-EB-DOTA-(PEG28)_2_-A20FMDV2 and [^177^Lu]Lu-IBA-DOTA-(PEG28)_2_-A20FMDV2 showed prolonged circulation half-life compared to the non-albumin binding [^177^Lu]Lu-DOTA-(PEG28)_2_-A20FMDV2. This led to significantly greater tumor inhibition, which was not present when using [^177^Lu]Lu-DOTA-(PEG28)_2_-A20FMDV2 at the same doses. Toxicity due to increased uptake in normal tissues remains a concern as it may lead to a narrower therapeutic index. It is anticipated that the K16R substitution that has been recently described would have a significant impact on reducing normal tissue uptake and therefore toxicity. Future studies will evaluate this substitution with the IBA albumin binder and ^177^Lu to determine if the therapeutic index has been improved.

## Data Availability

All data have been included in this article.

## Supplementary Materials

The following supporting information can be downloaded at: https://www.mdpi.com/article/10.3390/ph15020229/s1, Figure S1: Radio-TLC chromatograms in 50mM DTPA; Figure S2: Stability assays of [^177^Lu]Lu-EB-DOTA-(PEG28)_2_-A20FMDV2 and [^177^Lu]Lu-IBA-DOTA-(PEG28)_2_-A20FMDV2; Figure S3: Biodistribution of ^177^Lu radiotracers in CD-1 mice; Figure S4: Inhibition of BxPC-3 tumor growth and body weight changes following treatment with 37 MBq ^177^Lu]Lu-EB-DOTA-(PEG28)_2_-A20FMDV2 or [^177^Lu]Lu-IBA-DOTA-(PEG28)_2_-A20FMDV2; Figure S5: Hematoxylin-Eosin (H&E) and Ki-67 staining of kidney and tumor tissue slices collected for the control (saline) and 37 MBq [^177^Lu]Lu-IBA-DOTA-(PEG28)_2_-A20FMDV2 (*n* = 4); Table S1: Tumor-to-normal-organ ratios of [^177^Lu]Lu-EB-DOTA-(PEG28)_2_-A20FMDV2 in α_v_β_6_-positive BxPC-3 tumor-bearing mice; Table S2: Tumor-to-normal-organ ratios of [^177^Lu]Lu-IBA-DOTA-(PEG28)_2_-A20FMDV2 in α_v_β_6_-positive BxPC-3 tumor-bearing mice.
