# Supplementary material for: Therapeutic Efficacy of 177Lu-Labeled A20FMDV2 Peptides Targeting ανβ6
Source: Pharmaceuticals (Basel). 2022 Feb 15;15(2):229. doi: 10.3390/ph15020229 (PMC8876964; doi:10.3390/ph15020229)
Supplement: Supplementary file 1 [file pharmaceuticals-15-00229-s001.zip › pharmaceuticals-1585435-supplementary.pdf]

Supplementary Materials

Therapeutic Efficacy of ^{177}Lu-Labeled A20FMDV2 Peptides Targeting $\alpha_v\beta_6$

Truc Thao Huynh, Sreeja Sreekumar, Cedric Mpoy and Buck Edward Rogers

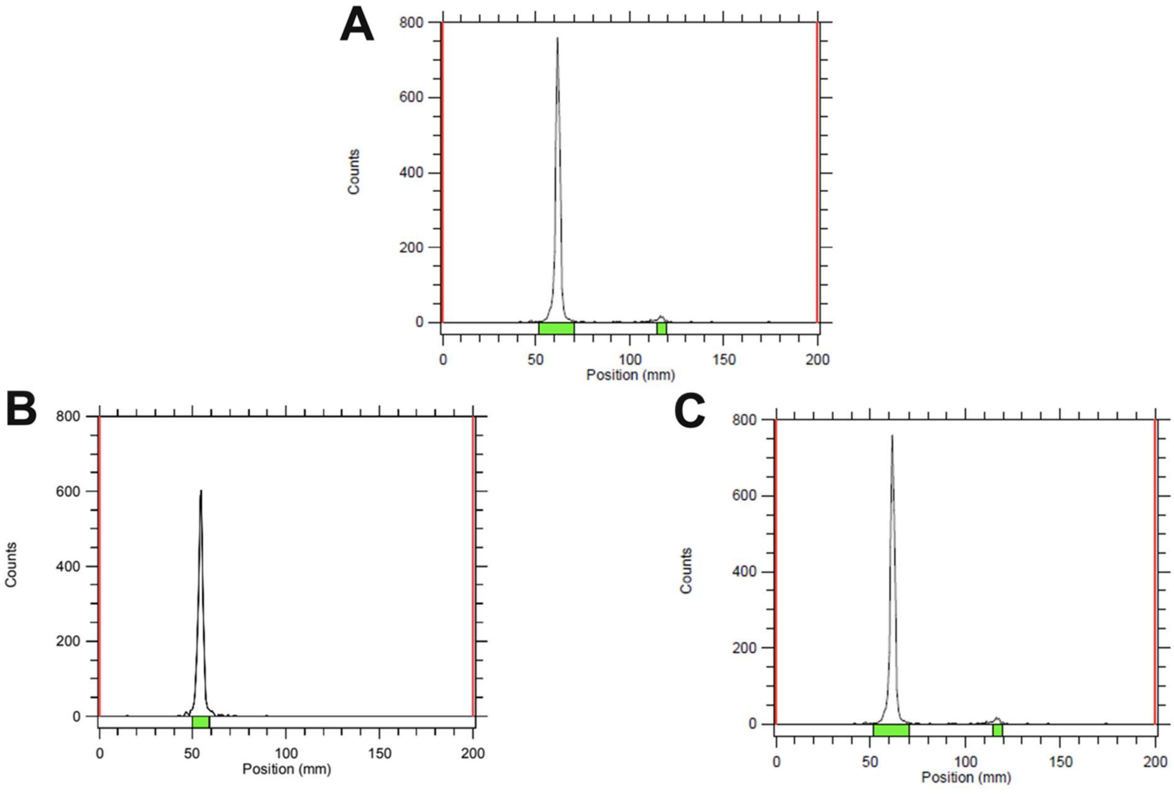

Figure S1 . Radio-TLC chromatograms in 50mM DTPA (A) [^{68}Ga]Ga-DOTA-(PEG28)$_2$-A20FMDV2 (B) [^{177}Lu]Lu-EB-DOTA-(PEG28)$_2$-A20FMDV2, and (C) [^{177}Lu]Lu-IBA-DOTA-(PEG28)$_2$-A20FMDV2.

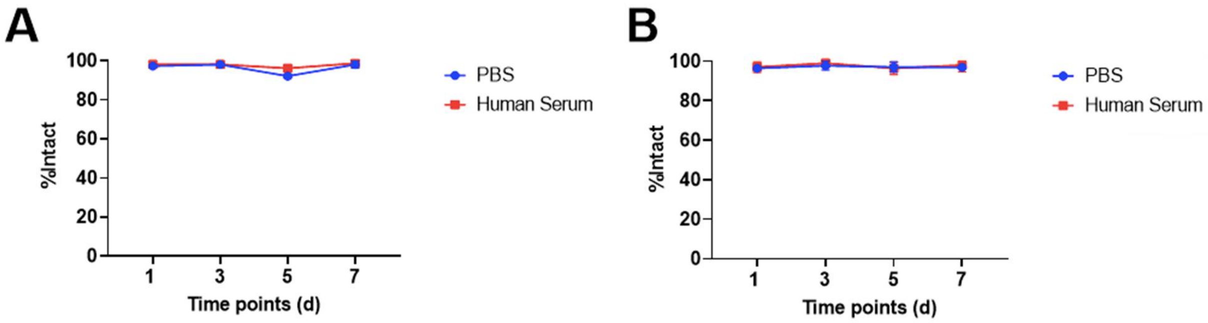

Figure S2 . Stability assays of (A) [^{177}Lu]Lu-EB-DOTA-(PEG28)$_2$-A20FMDV2 and (B) [^{177}Lu]Lu-IBA-DOTA-(PEG28)$_2$-A20FMDV2.

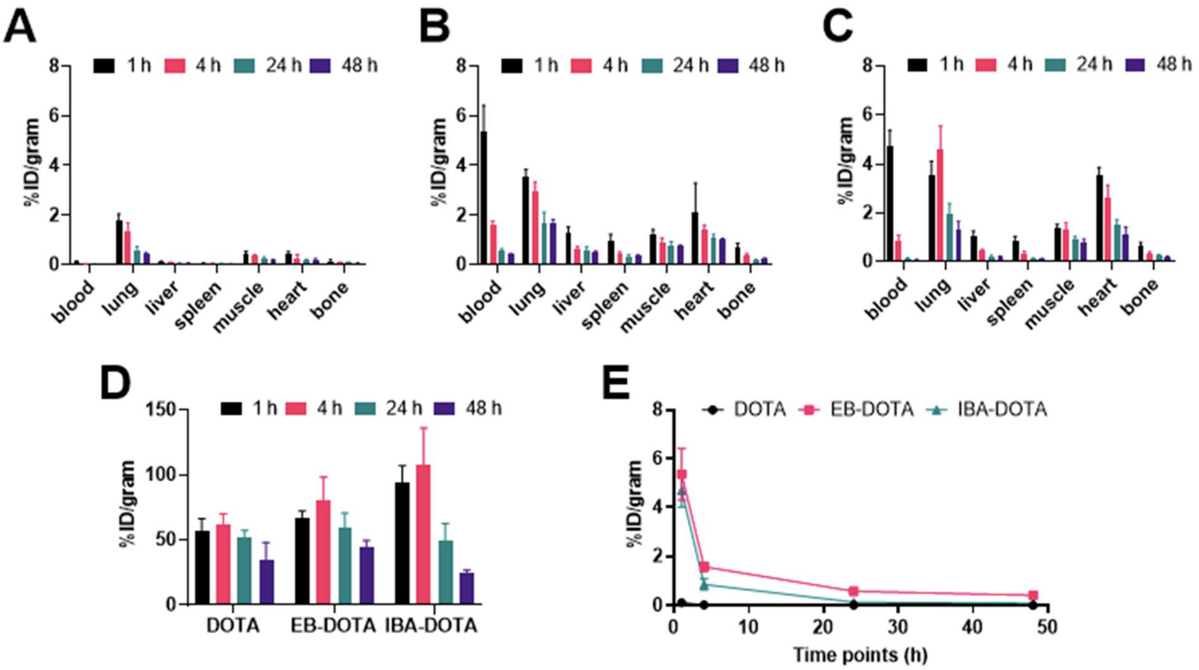

Figure S3 . Biodistribution of ^{177}Lu radiotracers in CD-1 mice (A) Radiotracer uptake of [^{177}Lu]Lu-DOTA-(PEG28)$_2$-A20FMDV2 (B) Radiotracer uptake of [^{177}Lu]Lu-EB-DOTA-(PEG28)$_2$-A20FMDV2 (C) Radiotracer uptake of [^{177}Lu]Lu-IBA-DOTA-(PEG28)$_2$-A20FMDV2 in tumors and selected organs (%ID/g; bars = SD; tumors: n= 4 – 5 per time point) (D) Kidney uptake of ^{177}Lu radiotracers (E) Blood clearance of ^{177}Lu radiotracers.

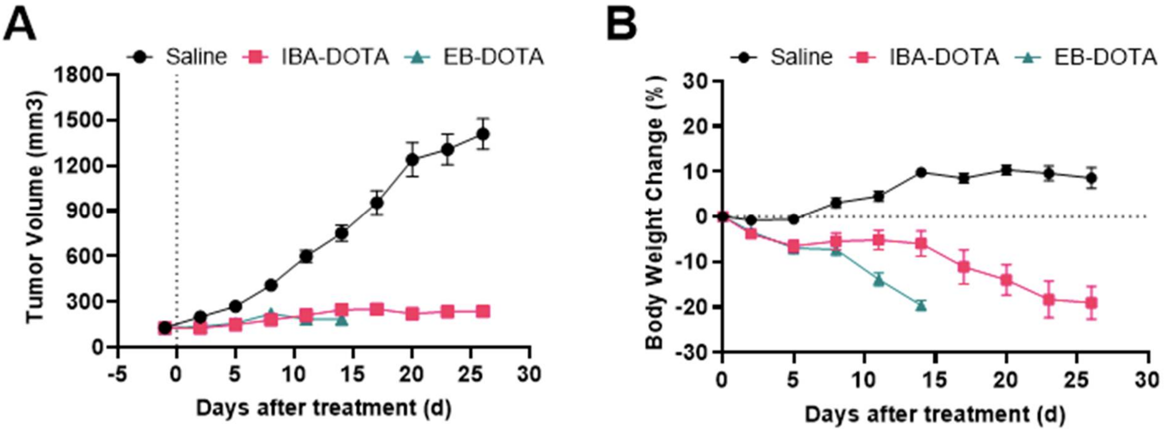

Figure S4 . Inhibition of BxPC-3 tumor growth and body weight changes following treatment with 37 MBq ^{177}Lu]Lu-EB-DOTA-(PEG28)2-A20FMDV2 or [^{177}Lu]Lu-IBA-DOTA-(PEG28)2-A20FMDV2 (A) Mean tumor volume (±SEM) (n = 8) with the dotted line indicating day of injection. Tumor volume was calculated as length (mm) × width (mm) × width (mm) / 2 (B) Body weight changes (±SEM) with the dotted line indicating baseline. The data represents the mean percent weight change from baseline (day 0) for each group.

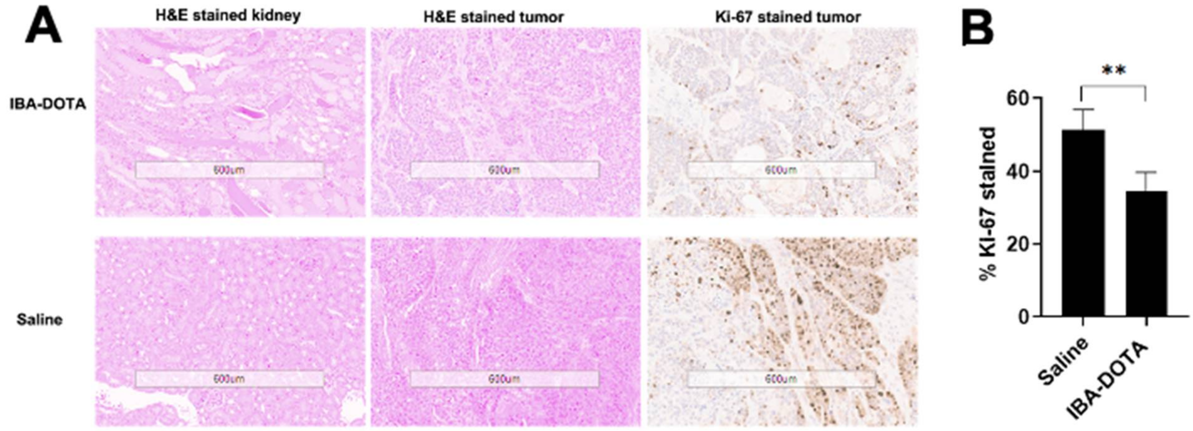

Figure S5 . Hematoxylin-Eosin (H&E) and Ki-67 staining of kidney and tumor tissue slices collected for the control (saline) and 37 MBq [^{177}Lu]Lu-IBA-DOTA-(PEG28)2-A20FMDV2 (n = 4) (A) Representative tissue slides taken under microscope at 4x magnification (B) Ki-67 proliferation index in tumors of the control and [^{177}Lu]Lu-IBA-DOTA-(PEG28)2-A20FMDV2 treated group (bars = SD, ** p < 0.01)

Table S1. Tumor-to-normal-organ ratios of [^{177}Lu]Lu-EB-DOTA-(PEG28)$_2$-A20FMDV2 in $\alpha_v\beta_6$-positive BxPC-3 tumor-bearing mice.

	1 h	4 h	24 h	48 h
**blood**	1.08 ± 0.13	5.19 ± 1.18	14.32 ± 2.22	15.45 ± 2.07
**lung**	1.68 ± 0.20	2.71 ± 0.30	3.08 ± 0.50	2.86 ± 0.46
**liver**	4.39 ± 0.72	9.70 ± 1.26	9.17 ± 2.14	8.63 ± 2.02
**spleen**	5.51 ± 0.91	17.04 ± 1.93	17.75 ± 2.52	14.57 ± 1.82
**kidney**	0.05 ± 0.01	0.05 ± 0.01	0.05 ± 0.01	0.06 ± 0.01
**muscle**	3.68 ± 0.81	5.50 ± 0.71	7.14 ± 0.84	6.07 ± 1.06
**heart**	2.19 ± 0.39	6.11 ± 0.72	7.71 ± 0.89	6.41 ± 1.35
**bone**	7.36 ± 2.63	25.29 ± 6.34	30.40 ± 6.44	28.93 ± 8.81

Table S2. Tumor-to-normal-organ ratios of [^{177}Lu]Lu-IBA-DOTA-(PEG28)$_2$-A20FMDV2 in $\alpha_v\beta_6$-positive BxPC-3 tumor-bearing mice.

	1 h	4 h	24 h	48 h
**blood**	1.14 ± 0.19	4.82 ± 0.69	49.49 ± 14.28	116.33 ± 30.94
**lung**	1.65 ± 0.14	1.93 ± 0.36	2.45 ± 0.85	3.54 ± 0.98
**liver**	5.53 ± 1.15	12.74 ± 1.91	24.65 ± 7.80	32.47 ± 7.20
**spleen**	6.19 ± 0.99	18.28 ± 3.93	43.09 ± 13.86	58.75 ± 17.44
**kidney**	0.06 ± 0.01	0.04 ± 0.01	0.06 ± 0.02	0.11 ± 0.03
**muscle**	3.36 ± 1.06	5.43 ± 1.52	9.98 ± 3.31	12.57 ± 2.25
**heart**	2.34 ± 0.53	6.81 ± 1.52	14.87 ± 5.29	19.04 ± 4.61
**bone**	7.11 ± 1.57	25.47 ± 12.22	65.83 ± 37.52	77.10 ± 37.53
